# Changing Epidemiology and Functional Outcomes of Inpatient Rehabilitation in Asian Traumatic Brain Injury Cases before and during the COVID-19 Pandemic: A Retrospective Cohort Study

**DOI:** 10.3390/life13071475

**Published:** 2023-06-29

**Authors:** Karen Sui Geok Chua, Hui Xuan Kwan, Wee Shen Teo, Ruo Xi Cao, Choon Pooh Heng, Rathi Ratha Krishnan

**Affiliations:** 1 Institute of Rehabilitation Excellence (IREx), Tan Tock Seng Hospital Rehabilitation Centre, Singapore 569766, Singapore; weeshen_teo@ttsh.com.sg (W.S.T.); ruoxi_cao@ttsh.com.sg (R.X.C.); rathi_ratha_krishnan@ttsh.com.sg (R.R.K.); 2 Lee Kong Chian School of Medicine, Nanyang Technological University, Singapore 636921, Singapore; m190147@e.ntu.edu.sg; 3 Yong Loo Lin School of Medicine, National University of Singapore, Singapore 117597, Singapore; 4 Clinical Research and Innovation Office, Tan Tock Seng Hospital, Singapore 308433, Singapore; choon_pooh_heng@ttsh.com.sg

**Keywords:** traumatic brain injury, head injury, rehabilitation, COVID-19, functional independence measure, inpatient, elderly, Asia

## Abstract

Background: This study aimed to compare acute injury and rehabilitation characteristics for traumatic brain injury (TBI) inpatients during the pre and post COVID-19 pandemic periods. Methods: A retrospective study of TBI inpatients between 1 April 2018 and 31 December 2019 (pre COVID-19 period), and 1 July 2020 and 31 March 2022 (post COVID-19 period) was performed to compare demographics, premorbid comorbidity, TBI characteristics, rehabilitation complications, admission and discharge functional independence measure (FIM^®^), length of stay and discharge status. Results: A total of 187 data sets were analyzed (82 pre COVID-19 and 105 post COVID-19). Post COVID-19 TBI inpatients were older by 11 years (pre COVID-19 mean 55 years vs. post COVID-19 mean 66 years, and *p* < 0.001), with 23% higher female inpatients (pre COVID-19 13.4% vs. post COVID-19 36.2%, and *p* < 0.001) and 25% higher presence of comorbidities (pre COVID-19 52.4% vs. post COVID-19 77.1%, and *p* < 0.001). In the post COVID-19 group, total discharge FIM (Td-FIM) was significantly lower by ~12 points (pre COVID-19 94.5 vs. post COVID-19 82, and *p* = 0.011), Td-FIM ≥ 91 was lower by ~18% (pre COVID-19 53.7% vs. post COVID-19 36.2%, and *p* = 0.017), and the need for caregivers increased by ~17% (pre COVID-19 68% vs. post COVID-19 85.4%, and *p* = 0.006) Conclusions: Our findings signal a demographic shift towards older, frailer TBI with lower functional independence levels post COVID-19.

## 1. Introduction

Traumatic brain injury (TBI) is a heterogenous disorder of major public health significance, with lifelong effects and large tolls on patients and their caregivers [1]. Globally, TBI have caused 8·1 million years lived with disability (YLD) corresponding to age-standardized rates of 111 (82–141) per 10,000, with falls and road injuries being the leading causes of TBI [1,2]. Rehabilitation plays an essential role in reducing disability and improving functional outcomes [1].

To date, the COVID-19 pandemic has claimed 6,877,230 lives and 688,715,476 confirmed cases have been reported since the first confirmed case in 12 January 2020 in Wuhan, China [3,4]. Singapore’s first confirmed case was reported on 23 January 2020 [5]. The COVID-19 pandemic had upended healthcare systems and disrupted patient care in terms of referral patterns, neurosurgical management and neurorehabilitation services due to the measures undertaken to detect, contain and reduce viral spread [6,7,8,9,10].

Literature relating to TBI epidemiology, neurosurgical practice and rehabilitation revealed alterations in disease patterns during the initial and critical phases of the pandemic. Several countries had reported 25–60% reductions in road traffic accident (RTA) and sports-related TBI due to lockdowns, movement restrictions, working from home and school closures. Conversely, low-velocity falls in older patients and domestic violence related to assaults and alcohol use increased [11,12,13,14]. Mild TBI could also have been underreported during the pandemic, either because of not seeking or avoiding medical treatment due to fears of entering hospital or treatment at peripheral centers [11,12].

Regarding TBI neurosurgical management, some reports suggested that conservative management was preferred due to the predominance of mild and older TBI from low-velocity falls [14]. Other authors reported slight variations in neurotrauma practice with similar pre and post pandemic neurosurgical consultations, surgery rates, critical care admission needs and transfers to rehabilitation, despite significantly lower head trauma volumes by 28% [15,16]. These implied preparedness of these services during the pandemic, in contrast with other reports.

Worldwide, rehabilitation services in general, experienced major practice deviations during the COVID-19 pandemic, including early facilitated discharges, shortened stays, prioritized functional goals towards limiting physical impairment rather than improving activities or participation, and downsizing or closures of rehabilitation units [6,7,8,9,10]. Locally, inpatient rehabilitation services were provided at patients’ bedside rather than in rehabilitation gyms in order to limit staff movement. Daily encumbrances of donning of personal protective equipment and sanitization of gym equipment also reduced productivity and rehabilitation time. Disrupted patient–family interactions, carer training and communication ensued as a result of suspended or severely curtailed physical visits (30 min/day limited to two visitors at bedside) to hospitals [6].

Similarly, outpatient rehabilitation services were prioritized to patients’ acute medical needs in response to downsized capacities. In parallel, there was rapid deployment and acceptance of telemedicine and telerehabilitation services, in particular speech–language and psychological therapies [6,16].

Literature pertaining to the impact of the COVID-19 pandemic on TBI rehabilitation profiles and outcomes are unknown, in particular in regards Asian TBI [17]. We aimed to study the effects of the COVID-19 pandemic on inpatient TBI case-mix and rehabilitation outcomes by comparing two unique cohorts from similar duration pre and post pandemic periods.

The objectives of this study were to compare demographic, acute TBI and rehabilitation characteristics with regard to functional goal attainment, inpatient rehabilitation length of stay and discharge outcome between the pre and post COVID-19 periods. Secondly, we aimed to determine the factors correlating with rehabilitation outcome (discharge FIM) and independent attainment levels after inpatient rehabilitation.

## 2. Materials and Methods

### 2.1. Study Design

We conducted a retrospective review of the electronic medical records (EMRs) of patients who were admitted and discharged from inpatient TBI rehabilitation at a single tertiary center. Demographic, and TBI acute and rehabilitation functional data were independently extracted from a functional database registry which recorded prospective functional data during inpatient rehabilitation. Institutional ethical approvals were obtained from the National Healthcare Group-NHG Domain Specific Review Boards (NHG DSRB 2022/00168) prior to data extraction. Informed consent was waived as the study involved no human subjects and used only de-identified data.

### 2.2. Study Settings

The study was conducted at the Tan Tock Seng Hospital (TTSH) Rehabilitation Centre, a 95-bedded inpatient tertiary hospital with direct links to an acute neurosurgical unit and level II trauma center. The consultant-led inpatient TBI rehabilitation program consists of rehabilitation therapies conducted over 3 h daily and delivered 5.5 days a week by an interdisciplinary team of physiotherapists, occupational therapists, speech pathologists, nurses, social workers and psychologists, with regular sessional inputs from dieticians and orthotists. The daily TBI rehabilitation program consisted of management of disorders of consciousness, daily posttraumatic amnesia assessment and management, judicious neuropharmacology, mobility, balance and activities of daily living (ADL) practice, cognitive assessment and training, rehabilitation nursing and dietary interventions, TBI psychoeducation and discharge planning. The functional independence measure^®^ (FIM^®^) was recorded within 72 h of rehabilitation admission and discharge by FIM-certified rehabilitation therapists [18].

### 2.3. Study Participants and Study Period

EMRs based on inclusion criteria were patients aged >21 years, TBI confirmed by neurosurgeons and CT or MRI brain imaging and admitted within 12 weeks of date of TBI. We excluded EMRs of patients without a confirmed diagnosis of TBI (e.g., stroke, tumor, and infections), admitted >12 weeks from TBI onset and those with missing admission and discharge FIM data.

We compared 2 EMR data sets arising from 2 inpatient cohorts, collected over 21 months. The pre COVID-19 period referred to rehabilitation admissions between 1 April 2018 and 31 December 2019, while the post COVID-19 period referred to rehabilitation admissions between 1 July 2020 and 31 March 2022. Between 1 January 2020 and 30 June 2020, (this period was excluded from analysis) only 1 TBI patient was admitted because of severe disruptions to the local healthcare system due to Singapore’s “circuit breaker” from 7 April 2020 to 1 June 2020 [19]. This was effectively a national stay-at-home or lockdown order with severe population movement restrictions. Between March 2020 and September 2020, and July and September 2021 (both SARS-CoV-2-Delta subvariant surge), TTSH Rehabilitation Centre’s bed capacity reduced by 23% due to deployment of nursing staff to man COVID-19 wards.

### 2.4. Data Collection Procedures and Study Variables

EMRs were identified through the bed management unit and functional data from a rehabilitation standing database. Data extraction was performed independently without collecting personal identifiers and these data were used to construct a case record form consisting of independent variables and FIM data.

Independent variables included for analysis were demographic variables (age in years, sex—male/female, race—Chinese/non-Chinese, and pre morbid employment, including full-time, part-time or homemakers), presence of premorbid comorbidities, acute TBI characteristics (admission Glasgow Coma Scale (GCS), acute length of stay (ALOS) (days), ICU duration (days) and neurosurgical interventions (external ventricular drainage, decompressive craniectomy, clot evacuation, and ventriculoperitoneal shunting) [20].

The following premorbid comorbidities were searched for and analyzed: stroke, TBI, hypertension, hyper lipidaemia, diabetes mellitus, ischemic heart disease, end stage renal failure (ESRF), end stage liver failure (ESLF), chronic epilepsy, chronic smoking, chronic alcohol consumption, known psychiatric disorder, cancer or dementia.

Rehabilitation characteristics of importance included rehabilitation length of stay—RLOS (days), presence of motor impairment, and posttraumatic amnesia (PTA) defined as time from TBI till return of continuous memory of ongoing events (presence/emergence/duration in days) measured by rehabilitation professionals using the Westmead PTA scale (WPTAS) [21]. The WPTAS was used to assess PTA duration in days and PTA emergence for this study. It consists of 7 orientation items and 5 memory assessing prospective memory. The scale is administered on a daily basis and PTA emergence levels are achieved on the first of 3 days upon obtaining a score of 12/12 score or until the day of discharge from inpatient rehabilitation. At the first 12/12 score, the 3 memory picture cards are changed. The duration of PTA is calculated from the date of TBI till the first of the 3 consecutive daily scores of 12/12, denoting PTA emergence, or until the day of discharge, if not emerged [21]. For durations of >28 days of PTA, the first day of a 12/12 score was taken as the date of PTA emergence [22].

The presence of intra-rehabilitation complications was defined as those needing treatment or disrupting rehabilitation and further classified into medical or neurosurgical complications. A transfer-out to acute care due to either neurosurgical or medical deterioration, discharge disposition (home/community-hospital/nursing-home) and need for a caregiver were also analyzed.

Functional status during rehabilitation was measured using total FIM^®^ score ranging from 18 to 126 admission (Ta-FIM) and discharge (Td-FIM) across 18 domains—13 motor domains (sub-score 91) of self-care, sphincter-control, transfers, and locomotion and 5 cognitive domains (sub-score 35) of communication and social-cognition (FIM was recorded within 72 h of admission and discharge by certified therapists [18].

The primary outcomes for this study were total discharge FIM (Td-FIM) and functional independence levels as determined by Td-FIM of ≥ 91, wherein, FIM ≥ 91 indicated modified-to-independent levels, implying 6–7 points each, aggregated across the 18 domains. Secondary outcome measures included FIM gain (FIM-G) calculated as Td-FIM—Ta-FIM, and FIM efficiency (FIM-E) as FIM-G divided by RLOS (days) [18]. Both secondary outcomes quantify FIM absolute gain and FIM aggregated daily changes, respectively.

### 2.5. Statistical Analysis

Extracted data were exported to Microsoft Excel and subsequently to IBM Statistical Package for Social Sciences (IBM Corp, Armonk, NY, USA)) software version 28 for analysis.

Descriptive and summary statistics, mean, standard deviation, median and interquartile range were used. Factors analysis was performed to stratify likely factors correlating with Td-FIM and functional independence levels (Td-FIM of ≥91).

Both univariate and multivariate analyses were performed to determine factors correlating with Td-FIM and Td-FIM of ≥91. As data were non-normally distributed, comparison of differences between groups for ordinal data were conducted using the Mann–Whitney-U test, and comparison of differences for nominal data were analyzed with Pearson Chi square test.

Multiple variable linear regression for independent factors predicting Td-FIM was performed using the following variables: pre/post COVID-19 period, sex, employment status, rehab LOS, ICU stay (days), decompression craniectomy, clot evacuation, PTA emergence, transfer-out from rehabilitation and admission motor and cognitive FIM.

Binary logistic regression model for factors impacting discharge independence ability measured by Td-FIM ≥ 91 was performed using the following variables: pre/post COVID-19, employment status, rehabilitation LOS, decompressive craniectomy, clot evacuation, PTA emergence, presence of motor impairment, rehabilitation transfer-out, admission motor and cognitive FIM.

The level of significance for all tests was at *p* < 0.05.

## 3. Results

### 3.1. TBI Demographics and Acute Injury Characteristics

A total of 187 data sets were available for analyses, including 82 (43.9%) in the pre COVID-19 and 105 (56.1%) in the post COVID-19 period. There was a 28% (23/82) increase in the rehabilitation admissions post COVID-19.

Table 1 shows the comparison of demographic and acute TBI characteristics between the pre and post COVID-19 groups.

#### 3.1.1. TBI Demographics

Comparing pre and post COVID-19 samples, we found significant differences in age and sex distributions. Post COVID-19 TBI inpatients were older by 11 years (pre COVID-19 mean 55 years vs. post COVID-19 mean 66 years, and *p* < 0.001) and 23% more female TBIs was noted (pre COVID-19 13.4% vs. post COVID-19 36.2%, and *p* < 0.001). There were no significant differences in race (*p* = 0.079) nor employment status (*p* = 0.106).

#### 3.1.2. TBI Premorbid Comorbidities

A 25% increased prevalence of comorbidities (at least one) was found (pre COVID-19 52.4% vs. post COVID-19 77.1%, and *p* < 0.001). In particular, both cardiovascular and neurological comorbidities had increased significantly in the post COVID-19 population. Hypertension (pre COVID-19 29.3% (24) vs. post COVID-19 50.5% (53), and *p* = 0.003) increased by 1.7x in the post COVID-19 group, and stroke increased by 19 times (pre COVID-19 2.4% (2) vs. post COVID-19 45.7% (48), and *p* < 0.001). No significant changes in the prevalence of diabetes mellitus (*p* = 0.135), hyper lipidaemia (pre COVID-19 31.7% (26) vs. post COVID-19 53.3% (56), and *p* = 0.101) nor ischemic heart disease (*p* = 0.105) were found while comparing both groups.

The presence of dementia as well as psychiatric disorders increased ~10-fold (pre COVID-19 0% (0) vs. post COVID-19 9.5% (10), and *p* = 0.004) and ~6-fold (pre COVID-19 1.3% (1) vs. post COVID-19 7.6% (8), and *p* = 0.042), respectively. Presence of prior TBI increased by 17-fold in the post COVID-19 group (pre COVID-19 0% (0) vs. post COVID-19 17.1% (18), and *p* < 0.001).

#### 3.1.3. TBI Severity

TBI in general, was severe without significant between-group differences at a median PTA duration of >30 days with poor correlation between GCS severity classification where >50% were classified as mild (Table 1 and Table 3). The severity of TBI based on GCS level (Table 1) showed poor correlation with PTA duration with mild TBI (GCS 13–15) presenting with a median PTA duration of 64 days, moderate TBI (GCS 9–12) presenting with a mean PTA duration of 73 days and severe TBI (GCS 13–15) with a mean PTA of 85 days (*p* = 0.067).

#### 3.1.4. TBI Acute Management

Neurosurgical procedures decreased by 22% in the post COVID-19 group (pre COVID-19 58.5% (48) vs. post COVID-19 36.2% (38), and *p* = 0.002). Acute LOS was significantly shorter by 5 days in the post COVID-19 group (pre COVID-19 21 days vs. post COVID-19 16 days, and *p* = 0.004), with similar ICU admission rates of ~36% for both groups (*p* = 0.802).

The presence of neurosurgical procedures significantly correlated with longer ALOS, with operated patients staying >6 weeks longer prior to rehabilitation transfer compared to conservatively treated patients (LOS operated 119 days (86 cases) vs. LOS conservative 73 days (101 cases), and *p* < 0.001). Operated patients were also significantly younger by 6 years compared to the conservatively treated patients (age of operated 58 years (86 cases) vs. age of conservative 64 years (101 cases), and *p* = 0.027), thereby supporting conservative treatment of older TBI in the post COVID-19 group.

### 3.2. TBI Rehabilitation Outcome (FIM)

Table 2 shows the admission and discharge FIM total and subset scores.

Significant improvements in total, motor and cognitive FIM subset scores from admission to discharge were noted.

Table 3 shows the comparison of rehabilitation characteristics in the pre and post COVID-19 groups.

#### 3.2.1. TBI Rehabilitation Outcome (FIM)

In terms of rehabilitation outcome, admission total FIM (Ta-FIM) and FIM motor and cognitive sub-scores were similar in both groups (pre COVID-19 Ta-FIM 62 vs. post COVID-19 Ta-FIM 57, and *p* = 0.156). However, there was a significant decline in total discharge FIM (Td-FIM) by ~13 points in the post COVID-19 group (pre COVID-19 Td-FIM 95 vs. post COVID-19 Td-FIM 82, and *p* = 0.011), contributed predominantly by significant reductions Td-motor FIM (pre COVID-19 motor FIM 69 vs. post COVID-19 motor FIM 57, and *p* = 0.003), rather than Td-cognitive FIM subset scores which was similar between both groups (*p* = 0.387).

With regard to independence levels in the ability to attain FIM ≥ 91 at discharge, this was significantly lower by −17.5% in the post COVID-19 group (pre COVID-19 53.7% vs. post COVID-19 36.2%, and *p* = 0.017). In both groups, FIM-gain and FIM-efficiency were similar with medians of +25 points/episode of stay (*p* = 0.48) and +1.4 FIM points/day (*p* = 0.714), respectively.

#### 3.2.2. TBI Rehabilitation Characteristics

Overall rehabilitation LOS was similar in both groups at ~30 days (*p* = 0.6). There was an insignificant shortening of PTA duration by 5 days in the post COVID-19 group (pre COVID-19 36 days vs. post COVID-19 31 days, and *p* = 0.056), indicating similar TBI severities in both groups with similar proportions of PTA emergence (pre COVID-19 60.8% vs. post COVID-19 80.8%, and *p* = 0.3).

Overall, patients who experienced PTA after discharge had significantly shorter PTA durations by 20 days. (Emerged PTA duration 34 days, n = 113 vs. non-emerged PTA duration 54 days, n = 29, and *p* < 0.001).

#### 3.2.3. Discharge Function and PTA Status

PTA emergence was significantly correlated with higher Td-FIM scores by 22 points, indicating better independence with the resolution of PTA. (Td-FIM non-emerged 71 (SD 25.4) vs. Td-FIM emerged 93 (SD 22.2), and *p* < 0.0001). PTA emergence was also significantly correlated with a modified/independent status on discharge FIM≥ 91. (59% emerged attained independent level, (n = 113) vs. 21% non-emerged attained independent level (n = 33), and *p* < 0.001).

#### 3.2.4. Rehabilitation Discharge Status

The discharge home rates were similar in both groups (pre COVID-19 76.8% (63) vs. post COVID-19 73.3% (77), and *p* = 0.58), however, the need for a carer upon discharge was significantly higher in the post COVID-19 group (pre COVID-19 68.0% (55) vs. post COVID-19 85.4% (89), and *p* = 0.006). Overall, the need for carer on discharge was significantly associated with a lower Td-FIM by 36 points (carer needed Td-FIM 85 vs. no carer Td-FIM 121, and *p* < 0.001, and lower FIM ≥ 91 attainment by 22%, with 65% needing carer vs. 87% without carer needs, and *p* = 0.001).

#### 3.2.5. Rehabilitation Complications

The presence of intra-rehabilitation complications was similar in both groups (pre COVID-19 50% (41) vs. post COVID-19 56.2% (59), and *p* = 0.40) However, the rates of transfer-out of the rehabilitation unit for severe complications almost tripled in the post COVID-19 group (pre COVID-19 8.5% (7) vs. post COVID-19 24.8% (26), and *p* = 0.004). The majority of the increase was related to neurosurgical-related complications, which was ~3.8 times more in the post COVID-19 group (pre COVID-19 3.8% (3) vs. post COVID-19 14.3% (15), and *p* = 0.014), in comparison to similar percentages of medical transfer-outs between both groups (pre COVID-19 4.9% (4) vs. post COVID-19 13.4% (14), and *p* = 0.136).

#### 3.2.6. Factors Affecting Discharge FIM

Table 4 shows the multiple linear regression analyses of factors impacting Td-FIM.

Post rehabilitation functional independence (Td-FIM) was significantly negatively correlated with the post COVID-19 period, need for clot evacuation rather than decompressive craniectomy, longer ICU and rehabilitation stays; the latter two factors were of weak impact. Discharge function (Td-FIM) was positively correlated with male sex, premorbid employment, PTA emergence upon discharge from rehabilitation and higher motor and cognitive FIM; the latter two were of weak impact. These factors accounted for ~65% of the variance (*p* = 0.0001).

#### 3.2.7. Factors Affecting FIM Independence Level

Table 5 shows the logistic regression analysis of factors impacting the ability to be independent post discharge from rehabilitation (FIM ≥ 91).

Premorbid employment (OR 1.8, and *p* = 0.001), PTA emergence (OR 2.1, and *p* = 0.002) and motor FIM (OR 0.064, and *p* = 0.002) were significant protective factors in the attainment of functional independence levels at rehabilitation discharge. The post COVID-19 period (OR 0.588, and *p* = 0.247), cognitive FIM (OR 0.006, and *p* = 0.848), need for clot evacuation (OR −0.342, and *p* = 0.620), age nor comorbidity were not shown to have significant impact on independence level. Although not shown in the regression models, patients with at least one comorbidity did much worse in Td-FIM by 18 FIM points (comorbidity present Td-FIM 76 vs. comorbidity absent Td-FIM 94, and *p* ≤ 0.001).

## 4. Discussion

### 4.1. TBI Demographics and Acute Characteristics

This study aimed to quantify differences in demographic, acute TBI and rehabilitation functional characteristics and discharge outcome between two groups of TBI inpatients during similar pre COVID-19 and post COVID-19 periods. A 28% increase in rehabilitation admissions post COVID-19 was noted. Steep rises in TBI by as much as 117% had previously been reported in the early phases of the COVID-19 pandemic due to lifting of lockdowns or movement restrictions, excessive vehicular speeds and dangerous driving on uncongested roads [16,23,24,25,26,27].

Locally, the increase in TBI rehabilitation admissions could be explained by accelerated transfers of patients to the inpatient rehabilitation unit to free-up acute hospital beds to treat COVID-19 patients. The shorter ALOS for post COVID-19 compared to pre COVID-19 by 5 days as well as fewer patients needing acute surgeries due to milder TBI in older patients, supports this fact. Older TBI patients traditionally transferred to community hospital were now diverted to tertiary rehabilitation wards due to these community hospitals’ redeployments as COVID-19 transitional care facilities.

We noted significant changes in demographic profiles between pre and post COVID-19 groups. Post COVID-19 TBI were older by more than a decade, with a higher proportion of females, and higher incidence of cardiovascular and stroke comorbidities, both known associations with older age. The exact reasons for this difference are unclear. One of the possible reasons could be related to the current global demographic shift in TBI population towards increasing geriatric TBI and predominant etiology of falls, overtaking motor vehicle accidents as the main cause of TBI [1,2]. From 2004 to 2015, a report from Singapore’s trauma registry reported a 3-fold increase in elderly patients admitted to hospitals, which is an increase from 17% to 50%. The median age of trauma patients, particularly females, has increased steadily, which is in line with the longer life expectancy of women in Singapore. Although the elderly population grew about 1% annually in Singapore, the number of seriously injured elderly patients increased at a rate of 16.5% every year [28,29]. Without local national TBI registry data, an increased TBI prevalence post COVID-19 would be speculative [23,24,25,26].

Early studies during the COVID-19 lockdown periods had documented worsened cardiometabolic health because of lack of activity, sedentary behavior, worsened dietary quality, increase in food intake and increased alcohol consumption [30,31].

The COVID-19 pandemic had also put the spotlight on mental health in general populations. The post COVID-19 cohort demonstrated significantly higher prevalence of psychiatric comorbidity and dementia on rehabilitation admission. Older age and higher comorbidities, in particular stroke, dementia and psychiatric disorders are known risk factors for TBI and with increasing age. Neuropsychiatric diseases and symptoms following COVID-19 infection, such as insomnia, depression, and anxiety, had been reported in patients, drawing parallels to previous viral pandemic-related outcomes [32,33]. Mental health patients without COVID-19 infection also defaulted outpatient follow-ups medications due to COVID-19 restrictions [2].

Regarding TBI acute severity, our data did not indicate significant differences between pre or post COVID-19 groups in terms of GCS, ICU admission rates and PTA duration. The entire sample’s mean age of 61 years was suggestive of an older TBI population and there was a poor correlation between GCS and PTA (*p* = 0.067). The GCS may not reliably predict mortality nor morbidity in older adults unlike younger TBI individuals due to unique neuropathology of geriatric TBI. The older patient may have evidence of expanding intracranial lesions on CT scans, often with a mild TBI (GCS 13–15) classification, leading to delayed clinical presentation and prolonged PTA duration [34,35].

Significantly lower neurosurgical rates were found post COVID-19 concurring with other studies on elderly TBI favoring a conservative approach due to increased operative risks and increased comorbidities. Management intensity of hospitalized TBI patients decreases with advanced age and low management intensity has been associated with an increased risk of 30-day mortality [28,34,35,36].

### 4.2. Rehabilitation Functional Characteristics

Findings from our study indicate that acute TBI inpatient rehabilitation facilitates recovery of functional independence [37]. Overall, significant improvements in total, motor and cognitive FIM subset scores from admission to discharge were noted for both groups, with similar FIM-G (Δ + 25) and FIM-E (Δ + 1.38) exceeding MCID reported for TBI [38]. These findings indicate that FIM gains translated to meaningful gains in patient’s lives after inpatient rehabilitation. In spite of multiple challenges in rehabilitation operations during the early pandemic period, a similar FIM gains were noted comparing pre and post pandemic samples. This was attributed to continued neurosurgical referrals, resilient inter-disciplinary rehabilitation teamwork and organizational preparedness.

Overall, Td-FIM was significantly worse in the post COVID-19 group, by −12 FIM points compared to the pre COVID-19 group, with 17.5% fewer patients attaining independence (FIM ≥ 91, and *p* = 0.017). Both multiple variable regression and binary logistic regression analysis identified three similar and significant factors: premorbid employment (+6 FIM, OR 1.8), PTA emergence (+12 FIM, OR 2.1) and a higher admission motor FIM as significant variables towards a higher Td-FIM score, and higher independence levels without care needs for FIM ≥ 91.

Higher motor FIM and return of continuous memory post PTA emergence imply better locomotor and motor and cognitive relearning capabilities for ADL independence and higher functional levels. Regarding the role of premorbid employment as a protective factor for higher Td-FIM, the current literature is non-informative on its exact mechanisms. Employed TBI patients may have higher cognitive reserves, educational levels and socioeconomic status influencing better functional outcomes [39]. Studies had pointed towards sociodemographic factors, such as premorbid unemployment, female sex, lower educational level and living alone, as associated factors for persistent health-related QoL impairments, poorer TBI outcomes and recovery [39].

That older age and comorbidities were not identified as significant correlates of poorer outcome related to Td-FIM or FIM ≥ 91 was unexpected. Various studies have reported an association between age, premorbid intelligence and years of formal education for mild to severe TBI impacting late cognitive recovery in the domains of memory, attention and executive function, lending support for the role of cognitive reserve and age in TBI cognitive recovery [39,40]. Another plausible reason was that of sampling error and preadmission selection bias of a small cohort of relatively young-old (mean age 61 years and oldest at 80 years) but still robust older population in spite of comorbidity.

There is also substantial evidence that intensive inpatient rehabilitation significantly benefits older adults with TBI, with the majority showing functional gains and getting discharged. Measurable post discharge functional improvements from older TBI undergoing inpatient brain injury rehabilitation programs are not dissimilar to younger patients, and older TBI people are able to live in community settings, although at higher costs, slower functional changes by half and longer hospitalizations stays by 2–3 times compared to younger individuals aged >55 years [41,42,43,44,45,46].

### 4.3. Correlates of Rehabilitation Outcome

That discharge motor FIM rather than discharge cognitive FIM was worse in the post COVID-19 group could possibly be explained by institutional responses to the pandemic resulting in practice changes, such as truncation of rehab goals to shorten rehab LOS due to resource limitation, reduced space and time for walking practice due to movement restrictions, inability to train for community integration due to outdoor restrictions and cessation of therapeutic weekend passes. There were no significant differences in cognitive FIM between both groups, despite the older age of post COVID-19 TBI group which could be explained by similar duration of PTA 36 days pre COVID-19 vs. 31 days post COVID-19 (*p* = 0.056) and similar PTA emergence, implying similar TBI severity between both groups.

PTA emergence was associated with +Δ22 higher Td-FIM related to the recovery of ongoing continuous memory translating into improved motor relearning, procedural memory influencing ADL and functional independence. However, a trend towards a shorter PTA duration by 5 days in the older post COVID-19 group was unexpected, as older TBI usually have poorer and slower cognitive recovery due to poorer memory attention and executive dysfunction, associated with hippocampal aging and white matter atrophy [40,46,47].

While males comprise majority of TBI globally, poorer TBI outcomes may be suggested in females [39,47]. However, our findings did not confirm sex nor age to be significant negative correlates of rehabilitation functional outcome, although the post COVID-19 group had a higher proportion of older female TBIs.

### 4.4. Rehabilitation Complications and Discharge Placement

Overall, >50% of the cohort suffered intra-rehabilitation complications without significant differences between the pre/post COVID-19 period, which is in line with high complication rates of 80% reported in a study with disorder of consciousness TBI patients, reflecting ongoing complexity of TBI multi-system involvements [48]. Neurosurgical-related transfer-outs during rehabilitation were significantly higher by 5-fold in the post COVID-19 group, likely due to their nature and need for acute stabilization and secondary surgical intervention. (Table 3)

In spite of the post COVID-19 group needing more care related to lower proportions of FIM ≥ 91 levels, the majority (75%), similar in both groups of moderate to severe TBI returned home post rehabilitation. This was likely explained by sociocultural reasons, such as strong Asian family support and the availability of live-in employed domestic carers.

### 4.5. Study Limitations

We highlight the following study limitations; retrospective design, from a single center with a small sample size which may limit generalizability, the absence of imaging data covariates for TBI lesion location and lack of long-term follow-up. We also could not determine the duration of comorbidities prior to TBI. Our study findings need to be replicated by larger studies to assess the true impact of COVID-19 on TBI outcomes to better advise the rehabilitation field and healthcare systems.

## 5. Conclusions

This study describes and quantifies changes in TBI case-mix and inpatient rehabilitation outcomes before and during the COVID-19 pandemic. Our findings signal a demographic shift towards older, frailer, comorbid TBI with lower functional independence levels post COVID-19 with reduced functional independence and higher care needs.

An interplay of factors involving altered acute TBI demographics and pandemic-response altered rehabilitation practice are possible explanations. In line with global TBI trends, characteristics important to this altered TBI case-mix include older, females with severe TBIs related to falls but showing slower and smaller functional improvements, and greater disability associated with altered post rehabilitation living situations [49,50]. Such shifts have potential implications to increase healthcare and socio-economic burdens for survivors, families and population health systems managing TBI.

## Institutional Review Board Statements

The study was conducted according to the guidelines of the Declaration of Helsinki, and approved by the Institutional Review Board (or Ethics Committee) of National Healthcare Group-NHG Domain Specific Review Boards (NHG DSRB 2022/00168, date of approval: 23 June 2022).

## Figures and Tables

**Table 1:** Comparison of demographic and acute TBI characteristics for the pre and post COVID-19 groups (n = 187).

Variable	Total (n = 187)	Pre COVID-19 (n = 82)	Post COVID-19 (n = 105)	*p*-Value
**Age**				
Age (years, mean (SD)	61.0 (19.0)	54.7 (21.2)	65.9 (15.4)	**<0.001 ^a^**
**Sex, n (%)**				
Male	138 (73.8)	71 (86.6)	67 (63.8)	**<0.001 ^b^**
Female	49 (26.2)	11 (13.4)	38 (36.2)	
**Race, n (%)**				
Chinese	152 (81.3)	62 (75.6)	90 (85.7)	0.079 ^b^
Non-Chinese	35 (18.7)	20 (24.4)	15 (14.3)	
Employed, n (%) n = 186 *				
No	86 (46.2)	32 (39.5)	54 (51.4)	0.10 6^b^
Yes	100 (53.8)	49 (60.5)	51 (48.6)	
**Pre-Injury Comorbidities, n (%)**				
Presence of at least 1 comorbidity	124 (66.3)	43 (52.4)	81 (77.1)	**<0.001 ^b^**
Previous Stroke	50 (26.7)	2 (2.4)	48 (45.7)	**<0.001 ^b^**
Previous TBI	18 (9.6)	0 (0.0)	18 (17.1)	**<0.001 ^b^**
Hypertension	77 (41.2)	24 (29.3)	53 (50.5)	**0.003 ^b^**
Hyperlipidemia	82 (43.9)	26 (31.7)	56 (53.6)	0.101 ^b^
Diabetes Mellitus	50 (26.7)	17 (20.7)	33 (31.4)	0.135 ^b^
Ischemic Heart Disease	19 (10.2)	5 (6.1)	14 (13.3)	0.105 ^b^
End Stage Renal Failure	1 (0.5)	0 (0.0)	1 (1.0)	0.376 ^b^
End Stage Liver Failure	1 (0.5)	0 (0.0)	1 (1.0)	0.376 ^b^
Chronic Epilepsy	5 (2.7)	1 (1.2)	4 (3.8)	0.276 ^b^
Psychiatric Disorder	9 (4.8)	1 (1.2)	8 (7.6)	**0.042 ^b^**
Dementia	10 (5.3)	0 (0.0)	10 (9.5)	**0.004 ^b^**
Cancer	18 (9.6)	4 (4.91)	14 (13.3)	0.052 ^b^
Chronic Smoker	21 (11.2)	4 (4.9)	17 (16.2)	**0.015 ^b^**
Chronic Alcoholism	20 (10.7)	7 (8.5)	13 (12.4)	0.399 ^b^
**Etiology of TBI**				
Road traffic accident	65 (34.7)	33 (40.2)	32 (30.5)	0.136 ^b^
Fall	111 (59.4)	44 (53.6)	67 (63.8)	
Others **	11 (5.9)	5 (6.0)	6 (5.7)	
Admission GCS, n (%), n = 183 *				
Mild (13–15)	110 (58.8)	42 (53.8)	68 (64.8)	0.068 ^b^
Moderate (9–12)	38 (20.3)	15 (19.2)	23 (21.9)	
Severe (3–8)	35 (18.7)	21 (26.9)	14 (13.3)	
**ICU admission, n (%)**				
Absent	68 (36.4)	29 (35.4)	39 (37.1)	0.802 ^b^
Present	119 (63.0)	50 (64.6)	66 (62.9)	
**Neurosurgery, n (%)**				
Performed	86 (46.0)	48 (58.5)	38 (36.2)	**0.002 ^b^**
Types of surgeries				
ICP monitor	32 (17.1)	17 (20.7)	15 (14.3)	0.245 ^b^
Decompressive craniectomy	24 (12.8)	15 (18.3)	9 (8.6)	0.049 ^b^
EVD	10 (5.3)	8 (9.8)	2 (1.9)	**0.018 ^b^**
Evacuation of clot	33 (17.6	11 (13.4)	22 (21.0)	0.142 ^b^
Craniotomy	27 (14.4)	9 (11.0)	18 (17.1)	0.234 ^b^
VP shunt	4 (2.1)	4 (4.9)	0 (0.00)	**0.022 ^b^**
Tracheostomy	7 (3.87	4 (4.9)	3 (2.9)	0.470 ^b^
**Post TBI seizure, n (%),**				
Present	20 (10.7)	6 (7.3)	14 (13.3)	0.187 ^b^
**ALOS (days), median (IQR)**	18.0 (14.0)	21 (15.3)	16 (11.0)	**0.004 ^a^**

^a^ Mann–Whitney U test; ^b^ Pearson Chi Square test. * Missing data. N = 187 unless otherwise stated. ** Others: 3 assaults, 2 sporting-related, 2 struck by object, 4 unspecified (n = 11). Legends: ICU—intensive care unit, TBI—traumatic brain injury, ICP—intracranial pressure, EVD—external ventricular drainage, VP shunt—ventriculoperitoneal shunt, ALOS—acute length of stay.

**Table 2:** Group summary table on inpatient admission and discharge FIM (n = 187).

Variables	Admission	Discharge	*p*-Value
Total FIM, median (IQR)	61.0 (39.0)	87.0 (40.0)	**<0.001 ^a^**
Motor FIM, median (IQR)	38.0 (28.0)	61.0 (32.0)	**<0.001 ^a^**
Cognitive FIM, median (IQR)	18.0 (17.0)	25.0 (11.0)	**<0.001 ^a^**

^a^ Wilcoxon Signed Rank test.

**Table 3:** Comparison of rehabilitation characteristics between pre and post COVID-19 groups (n = 187).

Variable	Total (n = 187)	Pre COVID-19 (n = 82)	Post COVID-19 (n = 105)	*p*-Value
PTA emergence, n (%) n = 146 *				
Absent	33 (22.6)	18 (24.1)	15 (19.2)	0.297 ^a^
Present	113 (77.4)	50 (60.8)	63 (80.8)	
**PTA Duration in days, median (IQR), n = 146**	32 (20.8)	36 (26.5)	31 (15)	0.056 ^b^
**Motor impairment, n (%)**				
Absent	150 (80.2)	67 (81.7)	83 (79.0)	0.651 ^a^
Present	7 (19.8)	15 (18.3)	22 (21.0)	
**LOS Rehab (days), median (IQR)**	30 (24.0)	29 (31.3)	30 (18.0)	0.598 ^b^
**Medical complications, n (%)**				
Present	100 (53.5)	41 (50.0)	59 (56.2)	0.400 ^a^
**Transfer out during rehab, n (%)**				
Present	33 (17.6)	7 (8.5)	26 (24.8)	**0.004 ^a^**
Neurosurgical **	18 (9.6)	3 (3.8)	15 (14.3)	**0.014 ^a^**
Medical	18 (9.6)	4 (4.9)	14 (13.4)	0.136 ^a^
**Discharge destination, n (%)**				
Home	140 (74.9)	63 (76.8)	77 (73.3)	0.584 ^a^
Institution/others	47 (25.1)	19 (23.2)	28 (26.7)	
**Carer needed, n (%)**				
Yes	144 (77.5)	55 (68.0)	89 (85.4)	**0.006 ^a^**
**FIM (admission)**				
Total FIM, median (IQR)	61 (39.0)	62 (44.3)	57 (35.5)	0.156 ^b^
Motor FIM, median (IQR)	38 (28.0)	40 (34.5)	37 (25.0)	0.120 ^b^
Cognitive FIM, median (IQR)	18 (17.0)	18 (16.0)	18 (18.0)	0.779 ^b^
**FIM (discharge)**				
Total FIM, median (IQR)	87 (38.0)	94.5 (37.3)	82 (39.0)	**0.011 ^b^**
Motor FIM, median (IQR)	61 (32.0)	69.0 (29.3)	57 (30.0)	**0.003 ^b^**
Cognitive FIM, median (IQR)	25 (11.0)	25.5 (11.3)	25 (11.0)	0.387 ^b^
**Calculated FIM scores**				
FIM gain, median (IQR)	25 (28.0)	25.5 (31.0)	23 (27.0)	0.477 ^b^
FIM efficiency, median (IQR)	1.38 (0.65)	1.36 (0.85)	1.39 (0.54)	0.714 ^b^
FIM ≥ 91, n (%)	82 (43.9)	44 (53.7)	38 (36.2)	
FIM <91, n (%)	105 (56.1)	38 (46.3)	67 (63.8)	**0.017 ^a^**

^a^ Pearson Chi Square test; ^b^ Mann–Whitney U test. N = 187 unless stated (* 41 (21.9%) of patients unable to undergo PTA assessment due to severe agitation, cognitive impairment or communication disorders). ** 18 including epilepsy (3), GCS deterioration (5), intracranial hemorrhage (5), syndrome of trephined brain (3) and hydrocephalous (2).

**Table 4:** Multiple linear regression analysis of factors impacting total discharge FIM (n = 187).

Variables	Multiple Linear Regression		
	Adj.coeff	95% CI	*p*-Value
**Post COVID-19** Pre/post (1 *)	−7.75	−13.31, −2.20	**0.007**
**Sex (male)**	6.36	0.69, 12.66	**0.048**
**Employment status**	10.69	5.083, 16.287	**0.0001**
**Rehab LOS**	−1.80	−0.310, −0.051	**0.007**
**Days of ICU stay**	−0.007	−0.012, −0.001	**0.014**
**Decompressive craniectomy**	−7.27	−15.343, 0.805	0.231
**Evacuation of clot**	−9.25	−16.341, −2.162	**0.011**
**PTA emergence by discharge**	11.84	−5.109, 18.573	**0.001**
**Transfer out during rehab**	−7.20	−14.889, 0.496	0.066
**Motor FIM on admission**	0.503	0.289, 0.716	**0.001**
**Cognitive FIM on admission**	0.47	0.094, 0.836	**0.014**

Variable selection stepwise method was used; R^2^ = 0.644. R^2^ is the percentage of total variance explained by the model. * Pre COVID-19 (0), post COVID-19 (1). Legend: LOS—length of stay, FIM—functional independence measure, PTA—post traumatic amnesia.

**Table 5:** Logistic regression analysis of factors impacting functional independence status level (FIM ≥ 91) (n = 187).

Variables	Binary Logistic Regression			
	B	Exp (B)	95% CI	*p*-Value
COVID-19 Pre/post (1 *)	−0.588	1.800	0.655, 4.868	0.247
**Employment status (1)**	1.785	4.193	0.062, 0.454	**0.001**
**LOS rehab**	−0.026	0.974	0.948, 1.000	0.053
**Decompressive craniectomy (1)**	0.992	2.698	0.546, 13.34	0.224
**Evacuation of clot (1)**	−0.342	0.710	0.184, 2.748	0.620
**PTA emergence upon discharge (1)**	2.112	0.121	0.032, 0.463	**0.002**
**Motor impairment present (1)**	−0.494	1.639	0.498, 5.397	0.417
**Transfer out during rehab (1)**	0.587	1.798	0.458, 7.054	0.400
**Motor FIM on admission**	0.064	1.066	1.024, 1.109	**0.002**
**Cognitive FIM on admission**	0.006	1.006	0.944, 1.000	0.848

* Pre COVID-19 (0), post COVID-19 (1) Legend: LOS—length of stay, FIM—functional independence measure, PTA—post traumatic amnesia.

## Data Availability

The data presented in this study are available on request from the corresponding author. The data are not publicly available due to institutional review board regulations.
